# *Cryptosporidium* infections in animals across Asia (2015–2025): a systematic review and meta-analysis of prevalence, host range, geographic distribution, and molecular epidemiology

**DOI:** 10.1186/s13567-026-01722-0

**Published:** 2026-04-28

**Authors:** Shahira Abdelaziz Ali Ahmed, Sonia Boughattas, Mohammad Reza Mahmoudi, Huma Khan, Simuzar Mamedova, Ardra Panavoor Namboodiri, Frederick R. Masangkay, Panagiotis Karanis

**Affiliations:** 1 Department of Parasitology, Faculty of Medicine, Suez Canal University, Ismailia, 41522 Egypt; 2 Institut Pasteur Tunis, Tunis El Manar University, Tunis, Tunisia; 3 Biomedicine Research Center, Trauma Institute, Guilan University of Medical Sciences, Rasht, Iran; 4 Department of Parasitology and Mycology, School of Medicine, Guilan University of Medical Sciences, Rasht, Iran; 5 Department of Microbiology, University of Swabi, Swabi, 23340 Khyber Pakhtunkhwa Pakistan; 6 Institute of Zoology, Ministry of Science and Education, Republic of Azerbaijan, Baku, 1001 Azerbaijan; 7 Department of Life Sciences, Khazar University, 1001 Baku, Azerbaijan; 8 Department of Science, Faculty of Health and Environmental Sciences, Auckland University of Technology, Auckland Central, Auckland, 1010 New Zealand; 9 Department of Medical Technology, Faculty of Pharmacy, University of Santo Tomas, 1008 Manila, Philippines; 10 Research Center for the Natural and Applied Sciences, University of Santo Tomas, 1008 Manila, Philippines; 11 University of Nicosia Medical School, 24005, CY-1700 Nicosia, Cyprus; 12 Medical Faculty and University Hospital, University of Cologne, Cologne, Germany; 13 UNIC Athens, No. 17, 29Th Street Elliniko, 167 77 Athens, Greece

**Keywords:** *Cryptosporidium*, Cryptosporidiosis, meta-analysis, oocysts, prevalence, genotype, Asia

## Abstract

*Cryptosporidium* spp. is a globally significant protozoan parasite with profound implications for animals, humans, and environmental health. Despite extensive research, its distribution, prevalence, and diversity across Asia remain fragmented. A systematic review and meta-analysis were conducted on studies published between 2015 and 2025, incorporating 329 investigations from 29 Asian countries and analyzing 142,484 animal specimens. Pooled prevalence was calculated, and molecular epidemiological data were synthesized to characterize species distribution and subtype diversity. The overall pooled prevalence of *Cryptosporidium* among  animals across Asia was 12%, with the highest prevalence reported in West Asia (19.8%). Infections were most frequent in livestock (13.1%), followed by wildlife (9.1%), reflecting the parasite’s expanding host range. Molecular analysis revealed the presence of > 74 species and 71 subtypes, with *C. parvum* (*n* = 93), *C. andersoni* (*n* = 55), *C. bovis* (*n* = 43), and *C. ryanae* (*n* = 40) being the most frequently reported. *C. parvum* demonstrated the broadest host spectrum, detected in > 37 animal species, with subtype family IId (n = 49) prevalent in > 22 hosts, underscoring its epidemiological importance. Over the past decade, the prevalence of *Cryptosporidium* and its host diversity has increased significantly across Asia, accompanied by marked genetic heterogeneity. These findings provide critical insights into transmission dynamics and regional hotspots. This synthesis highlights the urgent need for coordinated surveillance and a “One Health Strategy” to mitigate the risks posed by this adaptable and widespread pathogen.

**Supplementary Information:**

The online version contains supplementary material available at 10.1186/s13567-026-01722-0.

## Introduction

*Cryptosporidium* is a protozoan parasite of global concern that infects a wide range of vertebrate hosts, including humans, domestic animals, and wildlife. It causes cryptosporidiosis, a diarrheal disease with significant health implications, particularly in immunocompromised individuals and young children [1, 2]. While historically perceived as primarily a human health issue, current evidence underscores that *Cryptosporidium* is deeply rooted in ecological complexity, driven by interactions among multiple faunal assemblages and their shared environments. This interconnectivity forms the basis of a zoonotic transmission cycle that spans domestic, wild, and peridomestic animal populations, highlighting *Cryptosporidium* as a quintessential One Health pathogen [3–5].

Asia hosts nearly 60% of the global human population, the world’s highest livestock densities, and an unparalleled diversity of wild fauna, providing myriad ecological niches for *Cryptosporidium* [6]. It is a region of immense biodiversity, encompassing vast livestock population, diverse wildlife species, and rapidly urbanizing environments. These overlapping habitats facilitate frequent interactions among humans, animals, and contaminated environments, establishing a dynamic interface for *Cryptosporidium* transmission. The role of fauna in sustaining, spreading, and possibly evolving *Cryptosporidium* is multifaceted, with different host species contributing uniquely to the epidemiology of cryptosporidiosis [7].

Domestic animals, including cattle, sheep, goats, pigs, and poultry, are considered significant reservoirs of the zoonotic *Cryptosporidium* species. For instance, *C. parvum*, commonly detected in pre-weaned calves, is one of the primary zoonotic *Cryptosporidium* species responsible for human infections. Molecular studies from China, India, and Thailand have reported a high prevalence of *C. parvum* and *C. bovis* in calves; however, zoonotic transmission to humans is well established for *C. parvum*, whereas *C. bovis* is generally regarded as host-adapted to cattle, with only limited molecular similarity to human isolates reported in specific epidemiological contexts [8–10]. Livestock waste management, particularly in peri-urban settings, plays a crucial role in contaminating water sources, especially when agricultural runoff or untreated animal faeces enter drinking water systems [11].

Wildlife, especially free-ranging and synanthropic species such as rodents, non-human primates, deer, and birds, are increasingly recognized as key players in the transmission ecology of *Cryptosporidium*. These species often inhabit areas adjacent to human settlements and agricultural zones, providing opportunities for both environmental and direct transmission. In Southeast Asia, *C. muris* and *C. viatorum*, typically associated with rodent and non-human primate hosts, have been detected in both human and environmental samples, underscoring their potential public health impact [12–15]. Moreover, wild animals serve as sentinel species, revealing the presence and persistence of *Cryptosporidium* in ecosystems often overlooked by conventional surveillance.

Captive and exotic animals, such as those in zoos or animal sanctuaries, have also been implicated in harboring *Cryptosporidium* species. Studies from urban zoological gardens in China and Malaysia have reported multiple genotypes in carnivores, primates, and herbivores, including zoonotic *C. parvum* and host-specific variants like *C. felis* in felines [16–20]. The artificial proximity of diverse animal species within such facilities creates a microcosm of cross-species transmission potential, often exacerbated by shared enclosures and contaminated water sources.

Companion animals, notably dogs and cats, are less commonly associated with zoonotic transmission, yet they may act as mechanical carriers or incidental hosts. *C. canis* and *C. felis*, while generally considered species-specific, have been occasionally identified in immunocompromised human patients, suggesting opportunistic zoonoses under particular circumstances [21]. With the increasing number of pet-owning households in Asia’s urban centers, their role in the broader epidemiological picture, although limited, cannot be discounted entirely.

Sporadic surveys illustrate the scale of the problem yet remain fragmented by host species and geography. In dairy systems, molecular surveys from Inner Mongolia reported prevalences up to 47% and the co-circulation of four species (*C. bovis*, *C. andersoni*, *C. ryanae*, and zoonotic *C. parvum* subtype IIdA19G1) in cattle of all age classes [22]. In comparison, a work in northeast Thailand identified *C. bovis* and *C. ryanae* in 13.5% of intensively managed dairy herds and linked infection to animal husbandry factors such as flooring and water trough design [23]. Outside domestic animals, recent rodent studies across Guangxi, Yunnan, and Hunan Provinces in China detected six distinct *Cryptosporidium* genotypes, including zoonotic *C. viatorum* and *C. muris*, highlighting the reservoir potential of small mammals that interface with human dwellings and croplands [15]. Even in highly controlled environments, such as the Beijing Zoo, molecular screening in 2024–2025 expanded the parasite’s known host range to include exotic carnivores, primates, and ungulates [24].

The environmental stage is pivotal in the lifecycle of *Cryptosporidium*. Oocysts, the infective form, are highly resistant to standard disinfection methods and can persist for months in moist environments. This environmental hardiness allows *Cryptosporidium* oocysts to accumulate in water sources, agricultural soils, and on fresh produce, creating sustained transmission routes [25]. Faunal contamination, resulting from faeces deposited in or near water bodies, is a significant contributor to waterborne outbreaks in both rural and urban settings [26].

Surface water systems across Asia have repeatedly tested positive for *Cryptosporidium* oocysts, with the parasite’s presence correlating strongly with upstream livestock density and wildlife activity. In regions of India and Bangladesh, high oocyst counts in river and irrigation waters have coincided with human infection peaks during the monsoon season, when runoff is most significant [27–29]. In such settings, the convergence of animal waste, untreated sewage, and poor water infrastructure forms an ideal backdrop for parasite amplification and human exposure.

Agricultural practices further complicate this interplay. The use of untreated animal manure as fertilizer introduces oocysts into crop systems [30], while free-grazing livestock and wildlife crossing agricultural fields can spread contamination across large areas. These realities point to an integrated ecological network wherein different faunal groups do not merely act as isolated hosts but as participants in an interconnected, self-sustaining transmission web.

Despite mounting evidence supporting the role of fauna in *Cryptosporidium* transmission across Asia, available data remain fragmented across disciplines and regions. Comparative assessment indicates marked regional differences in reported prevalence, which are likely influenced by variation in host species investigated, diagnostic methods employed, and the extent of molecular surveillance. Studies from East and South Asia more frequently report higher detection rates and greater variability in species diversity, reflecting intensive molecular characterization [22, 23], whereas data from Central and parts of Southeast Asia remain sparse. These disparities underscore the need for harmonized surveillance and cross-regional synthesis to define the faunal contribution to zoonotic risk across the continent.

This systematic review aimed to bridge that gap by comprehensively evaluating the prevalence, host diversity, regional distribution, and molecular epidemiology of *Cryptosporidium* in faunal populations throughout Asia between 2015 and 2025. Special emphasis was placed on the transmission interfaces that link fauna to human infection and environmental contamination, thereby contributing to a One Health-oriented understanding of this parasite’s ecology.

## Methodology

### Search strategies and data sources

This systematic literature review was conducted in accordance with the PRISMA guidelines, with the PRISMA checklist provided in Additional file 1. The methodological process encompassed predefined inclusion and exclusion criteria along with a structured assessment of potential bias, as outlined below.

To investigate the prevalence, geographical, and epidemiological patterns of *Cryptosporidium* spp. in animals across Asia, an extensive literature search was performed through the PubMed database. The search spans from February 6 to March 21, 2025. No language restrictions were imposed; however, a temporal filter was applied to capture studies published between January 1, 2015, and February 6, 2025, thereby encompassing a decade of pertinent research.

The search method was confined to title/abstract/keywords utilizing MeSH terms/keywords, with the *Cryptosporidium* and cryptosporidiosis keywords combined with each Asian country name independently, for a total of 50 Asian countries employing Boolean positional operators ("AND, OR"):

(*Cryptosporidium* OR cryptosporidiosis) AND (Yemen OR Vietnam OR Uzbekistan OR United Arab Emirates OR Turkmenistan OR Timor Leste OR Thailand OR Tajikistan OR Taiwan OR Syria OR Sri Lanka OR Republic of Korea OR South Korea OR Singapore OR Saudi Arabia OR Russia OR Qatar OR Philippines OR Palestine OR Pakistan OR Oman OR Korea OR Nepal OR Myanmar OR Mongolia OR Maldives OR Malaysia OR Lebanon OR Laos OR Kyrgyzstan OR Kuwait OR Kazakhstan OR Jordan OR Japan OR Israel OR Iraq OR Iran OR Indonesia OR India OR Georgia OR Cyprus OR Cambodia OR Brunei OR Bhutan OR Bangladesh OR Bahrain OR Azerbaijan OR Armenia OR China OR Afghanistan). The search strategies and the applied MeSH keywords are presented in detail (Additional file 2). The reference lists of the included studies were also used to search for related/retrieved articles.

### Eligibility criteria and data extraction

Articles with titles that suggested a focus on *Cryptosporidium* or cryptosporidiosis in any animal population in the Asian continent were screened for eligibility and considered for inclusion in the literature review. Abstracts, along with potentially relevant full texts, were independently assessed by six authors (SA, MM, HK, SM, SB, and AN). Any discrepancies in selection were resolved through consensus involving three reviewers (SA, SB, and FM).

For the comprehensive full-text evaluation, a wide range of variables was systematically extracted, including: study location, year of publication, diagnostic methods employed for *Cryptosporidium* detection, animal kingdom categorization, taxonomic class, classification by animal use, animal type as reported in each article, age (when specified), clinical status or symptomatology (where available), number of infected animals, total sample size, and the overall prevalence of *Cryptosporidium* infection, either as stated by the authors or derived from the reported data. Additional details included methodological approaches, specific techniques applied, and the identified *Cryptosporidium* species, genotypes, and subtypes, when such information was provided.

Publications were excluded based on several criteria: absence of an abstract or full text, use of languages other than English, lack of relevance to the study’s objective, a primary focus on diagnostics or interventions, studies conducted outside the Asian continent, or a focus on genetic and/or protein analyses. Additional exclusions included articles unrelated to *Cryptosporidium*, review articles, case reports, commentaries, letters to the editor, multicenter studies, modeling or experimental studies, and research centered on humans, food, water, or soil. Studies were also excluded if they presented unclear or ambiguous data, lacked proper citation quality, or duplicated findings already published by the same author.

### Data analysis

The present review encompassed a comprehensive dataset, integrating multiple key variables: (a) the total number of included studies, (b) the number of Asian countries represented, (c) the number of distinct animal populations examined, and (d) the classification of animal populations.

To facilitate a clearer understanding of the geographical distribution of data across Asia, the continent was initially segmented into five major regions: Central, West, South, East, and Southeast Asia, following the regional classification outlined in the World Atlas [31].

In alignment with the UNSD [32], the analysis considered a total of 50 Asian countries (Afghanistan, Armenia, Azerbaijan, Bahrain, Bangladesh, Bhutan, Brunei, Cambodia, Georgia, China, Cyprus, Iraq, Iran, Indonesia, India, Israel, Japan, Jordan, Kazakhstan, Kuwait, Kyrgyzstan, Laos, Lebanon, Malaysia, Maldives, Mongolia, Myanmar, Nepal, North Korea, Oman, Pakistan, Palestine, Philippines, Qatar, Russia, Saudi Arabia, Singapore, South Korea, Sri Lanka, Syria, Taiwan, Tajikistan, Thailand, Timor-Leste, Turkey, Turkmenistan, United Arab Emirates, Uzbekistan, Vietnam, and Yemen).

The studies included in this review about the kingdom *Animalia* were systematically categorized as follows:

i. Broad kingdom classification: Organisms within the kingdom *Animalia* were first broadly classified into vertebrates and invertebrates.
ii. Taxonomic class classifications: For vertebrates, further classification was conducted according to taxonomic class, comprising: *Mammalia*, *Aves*, *Reptilia, Actinopterygii, Arthropoda* (e.g., flies), *Porifera* (e.g., sponges), and *Mollusca* (e.g., bivalves).
iii. Classification by animal use: Animals were classified further based on their functional use or living context:

- Wildlife: Animals living freely in their natural habitats.
- Companion: Domesticated animals kept as pets, for emotional support, as service animals, or used in laboratory research.
- Livestock/Work: Animals raised for agricultural production, food, or utilized for labor and/or transport.
- Captive animal facility: this category includes zoos, captive wild animal farms, ornithological gardens, state museums, conservation centers, tropical gardens, archaeological reserves, research centers for animal reproduction, national nature reserves, breeding bases of experimental animals, shelters, experimental animal rearing facilities, laboratory animal facility, rare wildlife rescue breeding centers, and wildlife parks.
- Stray: free-roaming animals found in urban environments without direct human ownership.
- Mixed: Populations comprising animals of varied uses (e.g., wild and household, or zoos, farms, free-range, or research laboratories).

A meta-analytical approach was employed to assess the prevalence and incidence of *Cryptosporidium* spp. across various Asian countries and among different animal populations or sample categories. Data were extracted from studies that met the predefined eligibility criteria, and a subgroup analysis was conducted to account for heterogeneity and diversity in target parameters (Ben Ayed et al., 2024). Statistical analyses were conducted using MedCalc software version 23.2.1 (MedCalc Software Ltd., Ostend, Belgium) to perform a meta-analysis of proportions and to estimate effect sizes and their corresponding 95% confidence intervals (CIs). Heterogeneity among studies was evaluated using Cochran’s Q statistic, with significance determined at *p* < 0.0005 based on a chi-square distribution. Potential publication bias was assessed using Egger’s and Begg’s tests, where a low *p*-value was indicative of bias in the published literature.

## Results

### Search results

From the PubMed database, a total of 1838 articles were initially identified. Following the removal of 339 duplicate records, 1499 titles and abstracts were screened. Of these, 398 publications were considered suitable for full-text evaluation. After a detailed eligibility assessment, 329 original studies met the inclusion criteria and were incorporated into the meta-analysis. The overall selection procedure, along with the corresponding flow diagram of the literature search, is provided in Additional file 3.

### Characteristics of the included studies

The studies incorporated into this analysis were published between 2015 and the first quarter of 2025, representing 58% (29 out of 50) of the countries across the Asian continent. No eligible studies were identified from the following countries: Afghanistan, Armenia, Bahrain, Bhutan, Brunei, Cambodia, Georgia, Kazakhstan, Kyrgyzstan, Laos, Lebanon, Maldives, Myanmar, North Korea, Oman, Singapore, Tajikistan, Timor-Leste, Turkmenistan, Uzbekistan, and Yemen.

When categorized by animal use, livestock accounted for most studies (*n* = 254), followed by companion animals (*n* = 64), wildlife (*n* = 62), captive facility animals (*n* = 50), stray animals (*n* = 16), and mixed-use populations (*n* = 16).

Regarding age distribution, 221 studies reported animal age, ranging from less than 1 month to over 10 years. In contrast, 108 studies lacked this information, creating a substantial gap in demographic characterization. A similar deficiency was evident in reporting clinical status: while 44 studies investigated symptomatic animals, 83 focused on asymptomatic populations, and 9 encompassed both groups, a majority of 193 studies failed to specify the clinical condition. Among symptomatic cases, the most frequently documented signs included diarrhea, anorexia, lethargy, blindness, epithelial lesions, abdominal distension, gill deformities, tremors, sunken eyes, feeding or drinking difficulties, inability to stand, regurgitation, gastric bulging, and, in severe instances, mortality.

### Features of the prevalence proportion of *Cryptosporidium* spp. in animals across Asia

#### Geographical *Cryptosporidium* prevalence in animals across Asia

Based on the data extracted from 329 studies, encompassing a total of 142,484 animal specimens, the pooled prevalence of *Cryptosporidium* spp. was estimated at 12% (95% CI: 10.6–13.4; Q = 21,571.2668; *P* < 0.0001; I^2^ = 98.4%) (Additional file 4).

**Figure 1:**
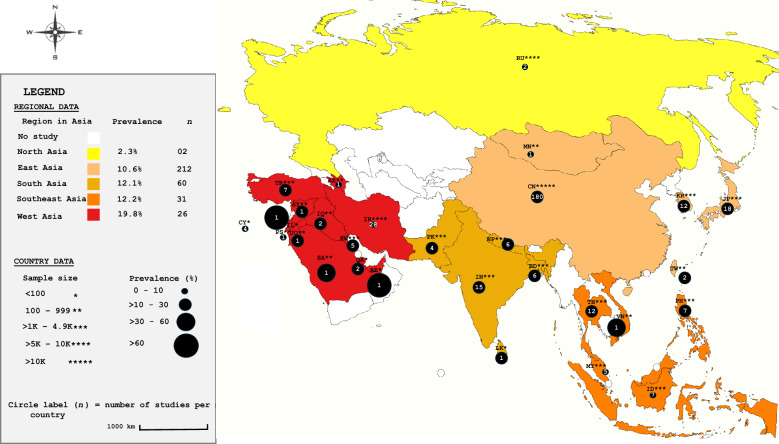
**Geographical distribution of *Cryptosporidium* spp. prevalence in animals across Asia (2015–2025).** Country code follows the Alpha-2 code of the country ISO codes as described in the ISO 3166 international standard (https://www.iban.com/country-codes). The order of country is arranged from highest to lowest prevalence: *IL* Israel, *AE* United Arab Emirates, *SA* Saudi Arabia, *VN* Vietnam, *JP* Japan, *PK* Pakistan, *PH* Philippines, *TR* Turkey, *IN* India, *SY* Syria, *TW* Taiwan, *IQ* Iraq, *QA* Qatar, *KW* Kuwait, *TH* Thailand, *JO* Jordan, *LK* Sri Lanka, *KR* South Korea, *NP* Nepal, *BD* Bangladesh, *CN* China, *MY* Malaysia, *AZ* Azerbaijan, *CY* Cyprus, *ID* Indonesia, *IR* Iran, *RU* Russia, *PS* Palestine, *MN* Mongolia. *n*, number of studies per region.

In this review, Asia was stratified into five regions: East, North, South, West, and Southeast Asia, with no eligible studies retrieved from Central Asia. A clear geographical gradient was observed, with the highest pooled prevalence recorded in West Asia (19.8%; CI: 18.8–20.8). This was followed by Southeast Asia (12.3%; CI: 11.5–13), South Asia (12.1%; CI: 11.6–12.6), and East Asia (10.6%; CI: 10.4–10.8), while North Asia exhibited the lowest prevalence at 2.4% (CI: 2–2.7) (Figure 1, Additional file 4). These findings should be interpreted with caution, as the notably low prevalence reported for North Asia was derived from only two studies, both conducted in Russia. Interestingly, despite representing a single country, this region contributed a larger sample size (7585 specimens) than West Asia, the region with the highest estimated prevalence, which had a combined sample size of 6312.

To further elucidate country-level patterns, an additional analysis was conducted, as no evidence of publication bias was detected according to Egger’s test (*P* = 0.9922) and Begg’s test (*P* = 0.6242). Data drawn from 29 countries revealed substantial heterogeneity (Q = 3,232.2032; *P* < 0.0001; I^2^ = 99.1%), with pooled prevalence estimates ranging from as low as 0.6% (CI: 0.1–1.5) in Mongolia to as high as 92% (CI: 80.7–97.7) in Israel (Figure 1, Additional files 4–6).

Across the 29 Asian countries represented, the number of studies ranged from a single publication in Azerbaijan, Israel, Jordan, Mongolia, Palestine, Saudi Arabia, Sri Lanka, Syria, the United Arab Emirates, and Vietnam to 180 in China (Figure 1). These findings warrant cautious interpretation, as the interplay between prevalence rates, sample sizes, and study numbers presents diverse perspectives. From a prevalence standpoint, Israel reported the highest proportion, whereas Mongolia documented the lowest, approaching zero; notably, both estimates were based on a single study each. Sample size patterns added further contrasts: China, with the largest cumulative dataset of 94 945 specimens across 180 studies, ranked only 21^st^ in prevalence (10%), suggesting relatively low infection rates despite extensive sampling. Conversely, the strikingly high prevalence figures of 92%, 80.6%, and 40% reported in Israel, the United Arab Emirates, and Saudi Arabia, respectively, were derived from a single study in each country. While China and Iran contributed the most studies (180 and 28, respectively), neither was among the countries with the highest prevalence, ranking 21^st^ and 26^th^ respectively (Additional file 7).

**Figure 2:**
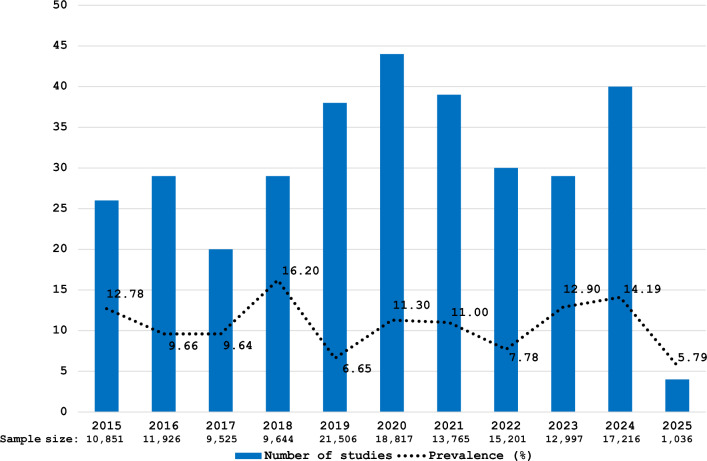
***Cryptosporidium* prevalence in animals across Asia by year of publication (2015–2025).**

When investigating temporal trends in the proportional prevalence of *Cryptosporidium* spp. among animals in Asia, as reflected in publications from 2015 to 2018, the number of studies showed no consistent increase or decline. Distinct surges in reported prevalence occurred in 2018 and again in 2024, marking the decade's most pronounced peaks (Figure 2). By contrast, only four studies were identified for the first quarter of 2025, suggesting the dataset for this period is likely incomplete and should be interpreted with caution.

#### *Cryptosporidium* spp. prevalence within the animal kingdom in Asia

**Figure 3:**
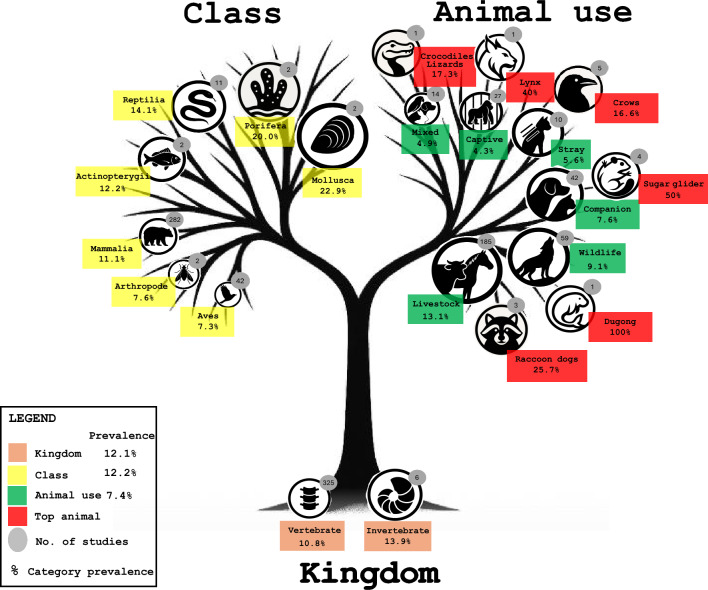
**Pooled prevalence of *Cryptosporidium* spp. in animals across Asia**. It is illustrated according to Kingdom, Class, and animal use categories. The size of each logo corresponds proportionally to its pooled prevalence, with larger logos representing higher values. Within each animal-use category, the species exhibiting the highest pooled prevalence (Top animal) is marked red. The logos and tree graphics were generated using Microsoft 365 Copilot based on prompts designed by F. R. Masangkay.

In this review, animal source data were derived from two kingdoms, invertebrates and vertebrates, totaling 142 484 samples (Q = 8.9832; *P* = 0.0027; I^2^ = 88.8%). While prevalence rates were broadly comparable between the two groups, an unexpected pattern emerged: invertebrates, represented by 974 specimens across six studies, exhibited a higher pooled prevalence of 13.9% (CI: 11.8–16.3). In contrast, vertebrates, despite contributing most of the data (141 510 specimens from 325 studies), showed a slightly lower pooled prevalence of 10.8% (CI: 10.6–10.9) (Figure 3, Additional file 4).

#### *Cryptosporidium* spp. prevalence within the animal classes across Asia

This review encompassed seven animal classes: Actinopterygii, Arthropoda, Aves, Mammalia, Mollusca, Porifera, and Reptilia. The highest prevalence was observed in Mollusca at 22.9% (CI: 18.8–27.4) based on 384 samples from two studies, followed by Porifera of 20% (CI: 6.8–40.7) based on 25 samples across two studies. By contrast, the lowest prevalence was observed in Aves at 7.4% (CI: 6.8–7.8), despite the comparatively large dataset of 12,119 specimens drawn from 42 studies (Figure 3, Additional file 4). Given the statistically significant heterogeneity (Q = 260.5148; *P* < 0.0001; I^2^ = 97.7%) and the absence of publication bias (Egger’s test, *P* = 0.9657; Begg’s test, *P* = 0.8806), a reliable random-effects subgroup meta-analysis was subsequently performed.

#### *Cryptosporidium* spp. prevalence within animal use in Asia

The analysis of *Cryptosporidium* contamination across Asian countries was stratified by animal use category (Q = 1,528.7597; *P* < 0.0001; I2 = 99.6%), encompassing captive facility animals, companion animals, livestock, mixed-use populations, stray animals, and wildlife. Livestock emerged as both the most extensively investigated group, comprising 86 033 specimens across 185 studies, and the category with the highest pooled prevalence at 13.2% (CI: 12.9–13.3). In contrast, the mixed-animal category, comprising on 5895 specimens from 14 studies, had the lowest prevalence at 5% (CI: 4.4–5.5) (Figure 3, Additional file 4).

##### Categorical prevalence of* Cryptosporidium* spp. within the captive animal facilities in Asia

**Figure 4:**
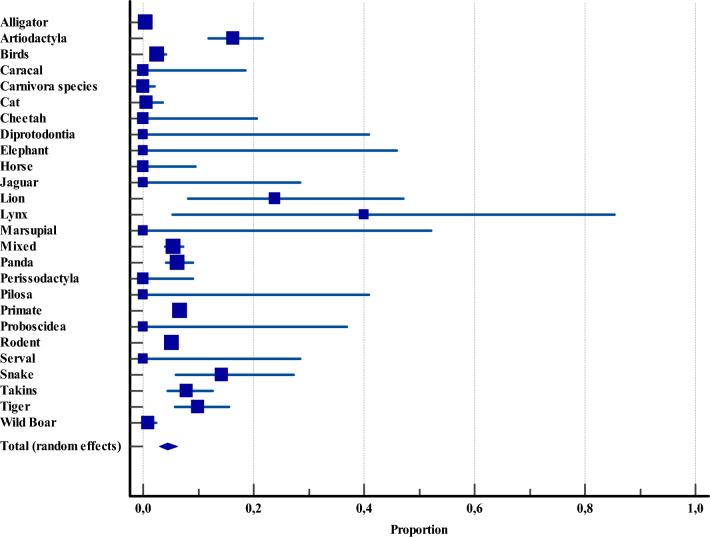
**Overall proportional prevalence of Cryptosporidium spp. within the captive animal facilities across Asia**.

Given the significant heterogeneity indices observed in the animal-use section, a categorical analysis was warranted. Within captive animal facilities (e.g., zoos, museums, conservation centers, ornithological gardens, archaeological reserves, experimental laboratories, breeding bases, and shelters), primates represented the most extensively studied group, with 3,166 specimens across 10 studies. However, they were not the most affected species. The highest prevalence of *Cryptosporidium* was reported in lynxes at 40% (CI: 5.2–85.3), followed by lions at 23.8% (CI: 8.2–47.1), and members of the order Artiodactyla at 16.3% (CI: 11.8–21.6). (Figure 4, Additional file 4).

##### Categorical prevalence of* Cryptosporidium* spp. within companion animals in Asia

**Figure 5:**
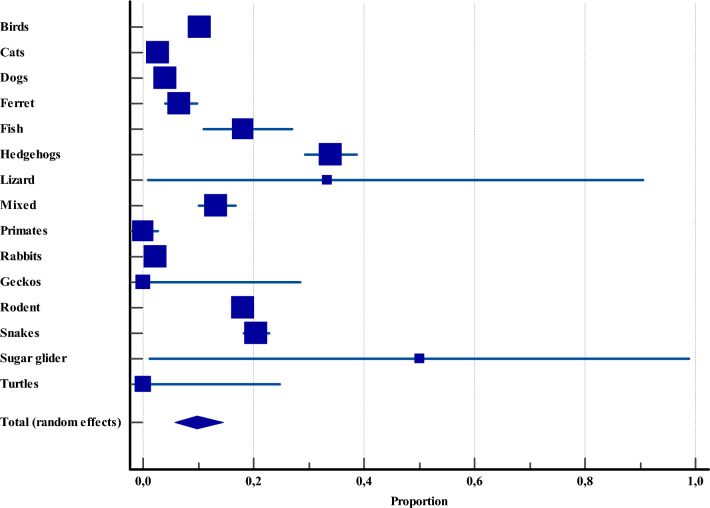
**Overall proportional prevalence of *Cryptosporidium* spp. within companion animals across Asia.**

The companion animals recorded in Asia and included in this analysis comprised birds, cats, dogs, ferrets, fish, hedgehogs, lizards, primates, rabbits, geckos, rodents, snakes, sugar gliders, turtles, and mixed-animal groups. Among these, dogs were the most extensively studied, with 8,088 specimens across 14 investigations. However, they were not the most heavily infected. Prevalence of *Cryptosporidium* among companion animals showed striking variability, ranging from as high as 50% (CI: 1.2–98.7) in sugar gliders to 0% in primates (CI: 0.0–2.6), geckos (CI: 0.0–28.4), and turtles (CI: 0.0–24.7) (Figure 5, Additional file 4).

##### Categorical prevalence of* Cryptosporidium* spp. within livestock in Asia

**Figure 6:**
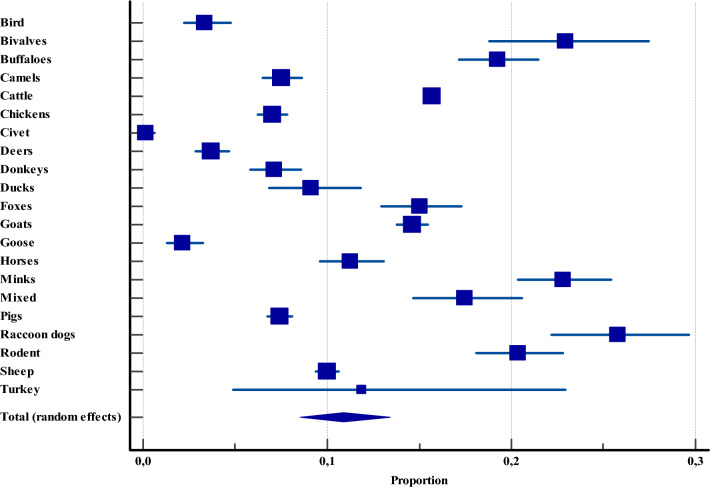
**Overall proportional prevalence of *Cryptosporidium* spp. within livestock across Asia.**

Among livestock in Asia, the prevalence of *Cryptosporidium* spp. displayed wide variation, ranging from as low as 0.1% (CI: 0.0–0.6) in civets to as high as 25.7% (CI: 22.1–29.6) in raccoon dogs. The second-highest prevalence was recorded in bivalves at 22.9% (CI: 18.8–27.4), followed closely by minks at 22.8% (CI: 20.3–25.4). Paradoxically, the species most extensively investigated: cattle (*n* = 43 469), sheep (*n* = 9277), and goats (n = 6,927), were not among the highest in prevalence, ranking only 7th, 12th, and 9th, respectively. Notably, these findings were consistent and not influenced by publication bias (Egger’s test, *P* = 0.1125; Begg’s test, *P* = 0.5065) (Figure 6, Additional file 4).

##### Categorical prevalence of* Cryptosporidium* spp. within stray animals in Asia

**Figure 7:**
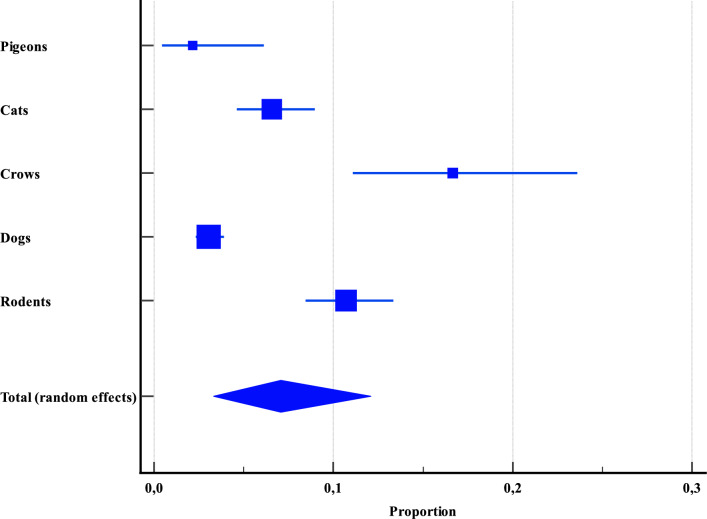
**Overall proportional prevalence of *Cryptosporidium* spp. within stray animals across Asia.**

The categorical analysis of stray animals in Asia encompassed 3398 specimens across 10 studies, yielding heterogeneous results (Q = 78.3803; *P* < 0.0001; I2 = 94.9%) with no evidence of publication bias (Egger’s test, *P* = 0.3154; Begg’s test, *P* = 1.0000). Remarkably, both the highest and lowest prevalence of *Cryptosporidium* within this group were reported in birds. Crows exhibited the highest prevalence at 16.7% (CI: 11.0–23.6), while pigeons showed the lowest at 2.1% (CI: 0.4–6.1) (Figure 7, Additional file 4). This contrast may be attributable to dietary differences, as crows are omnivorous, whereas pigeons primarily subsist on seeds and grains.

##### Categorical prevalence of* Cryptosporidium* spp. within the wildlife animals in Asia

**Figure 8:**
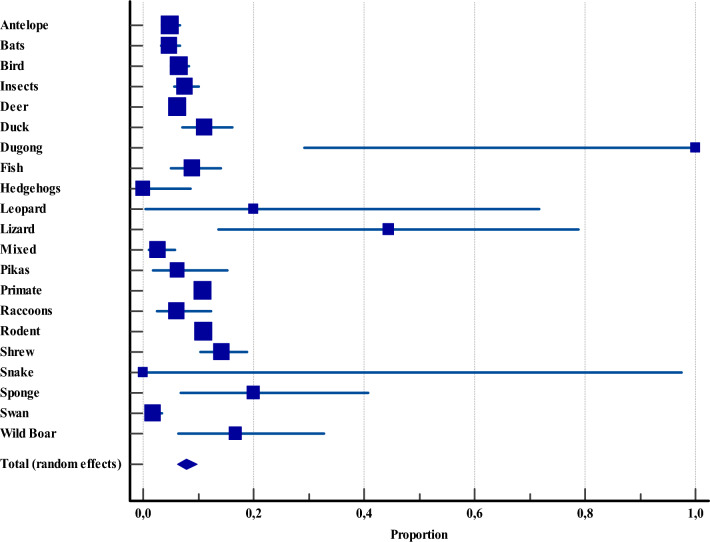
**Overall proportional prevalence of *Cryptosporidium* spp. within wildlife animals across Asia.**

This category encompassed 16 411 specimens from wild animals. Rodents were the most extensively studied group, with 6207 samples from 23 studies, while snakes were the least investigated, represented by a single specimen from a single study. Interestingly, snakes, along with hedgehogs, also exhibited the lowest prevalence of *Cryptosporidium* at 0% (CI: 0.0–97.5 and CI: 0.0–8.6, respectively). In contrast, the highest prevalence was observed in dugongs at 100% (CI: 29.2–100), followed by lizards at 44.4% (CI: 13.7–78.7). Notably, these exceptionally high prevalences were reported despite very minimal sample sizes, three specimens for dugongs and nine for lizards, each originating from a single study (Figure 8, Additional file 4). Importantly, no evidence of publication bias was detected (Egger’s test, *P* = 0.4823; Begg’s test, *P* = 0.1472).

##### Categorical prevalence of* Cryptosporidium* spp. within mixed animals in Asia

The mixed-animal category encompassed samples of unknown origin across 14 studies. This included birds (both domestic and wild pigeons), companions and stray animals (cats and dogs), and reptiles such as snakes from both wild and farmed sources. A separate mixed subgroup consisted of pooled specimens from domestic and stray dogs, as well as from cattle and water buffaloes.

**Figure 9:**
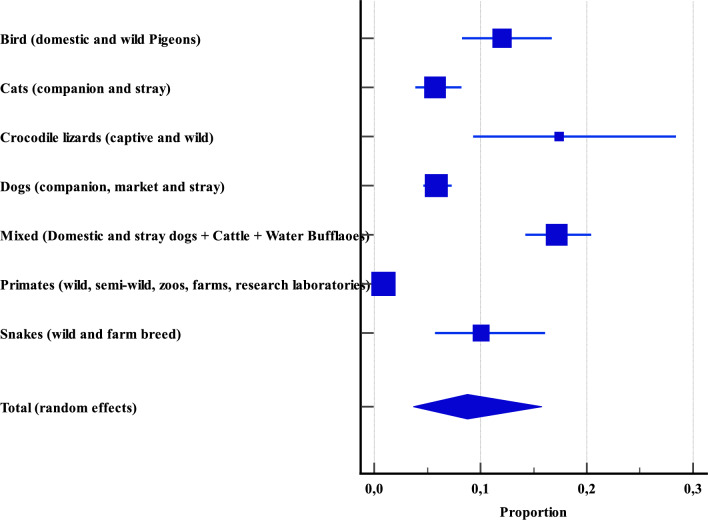
**Overall proportional prevalence of *Cryptosporidium* spp. within mixed animals across Asia.**

A total of 5895 specimens were examined. Among these, crocodile lizards, comprising both captive and wild individuals, demonstrated the highest prevalence of *Cryptosporidium* at 17.3% (CI: 9.3–28.4), despite being represented by only 69 samples. In contrast, primates, which accounted for the most extensive dataset with 3058 specimens (including wild, semi-wild, and captive orangutans along with other non-human primates from zoos, farms, free-range environments, and research facilities), exhibited the lowest prevalence at 0.8% (CI: 0.5–1.2) (Figure 9, Additional file 4).

The variations observed in the prevalence of *Cryptosporidium* spp. appear to be shaped by the taxonomic composition of the sampled animals, particularly at the levels of kingdom, class, and the functional role of animals. In the present dataset, vertebrates constituted the overwhelming majority of examined hosts, representing 99% (141 510/142 484), with mammals alone accounting for 89% (127 103/142 484). Notably, when stratified by animal use, livestock/work animals and companion animals were the dominant groups, comprising 86 127 specimens (prevalence 13.1%) and 21 205 specimens (prevalence 7.6%), respectively. Together, these categories encompassed 75% (107 332/142 484) of all animals tested, compared with the substantially smaller cohort of 29 351 animals represented by wildlife, strays, and captive animal facilities. Nonetheless, these findings warrant cautious interpretation, as shifts in the predominant animal categories under investigation could yield divergent prevalence patterns and alternative epidemiological outcomes.

### Applied methods and identified species/subtypes of *Cryptosporidium* in animals across Asia

#### Approaches for detecting *Cryptosporidium* in animal populations

In Asian studies, animal faecal samples were processed by applying concentration methods. Among these, sedimentation (formalin–ethyl acetate, formalin–ether) and flotation techniques, including “Fulleborn’s flotation” (saturated salt flotation, sugar flotation) were the most widely employed.

For the detection of *Cryptosporidium* spp., non-molecular, molecular, and combined methods were applied. Non-molecular techniques were applied in 51 investigations (15.1%, CI: 14.6–15.6), while molecular approaches were utilized in 213 studies (9.8%, CI: 9.6–10) in which a substantial proportion of the animals examined, representing 71% (101 025/142 484), were analyzed using molecular techniques, thereby ensuring a more standardized and reliable approach for the detection and genotyping of *Cryptosporidium* spp.

A combined application of both non-molecular and molecular techniques was reported in 65 studies (12.1%, CI: 11.6–12.4) for the identification of *Cryptosporidium* spp. infections (Additional files 4 and 8). Non-molecular detection of *Cryptosporidium* relied on classical parasitological and immunological methods. These included direct smears (wet-mount examinations with saline and iodine), staining procedures such as modified Ziehl–Neelsen (mZN), and a range of immunodiagnostic assays, including rapid immunoassays (RIA, e.g., immunochromatography), ELISA, and immunofluorescent assays (IFA).

Relative to molecular methodologies, a suite of PCR-based approaches was employed. Among them conventional PCR (cPCR), nested PCR (nPCR), restriction fragment length polymorphism PCR (RFLP-PCR), real-time PCR (qPCR), and sequencing strategies were utilized. The small subunit ribosomal RNA (SSU rRNA) gene was the predominant molecular target, with the *Cryptosporidium* oocyst wall protein (COWP) gene frequently serving as a secondary marker. Other loci, including actin and heat-shock protein 70 (Hsp70), were employed less often for species identification, while microsatellite markers (MS1, MS2, MS3, MS16, MS17, and MS18) were occasionally used to enhance genetic resolution. Subtyping efforts focused primarily on the glycoprotein 60 (*gp60*) gene, which remained the cornerstone for high-resolution genetic characterization of *Cryptosporidium* isolates.

From the initial 213 studies that used molecular approaches in *Cryptosporidium* spp. investigations, a total of 201 studies across Asia, examined the parasite at a genetic level. Eight studies used molecular tools for parasite identification only; two studies used next-generation approaches; and two studies reported null prevalence so no further molecular characterization steps were investigated (Additional file 9). Genotyping studies have documented a broad diversity of *Cryptosporidium* species. In some cases, a single species was identified, such as *C. parvum*, *C. hominis*, *C. meleagridis*, *C. felis*, *C. scrofarum*, or *C. suis*. Other investigations revealed multiple species within the same population, while certain studies detected mixed genotypes within individual animal isolates (Additional file 9).

#### Distribution frequencies of *Cryptosporidium* species/genotypes in animals across Asia

The analysis (Additional files 9–10) revealed a remarkable diversity of *Cryptosporidium* species and genotypes infecting animals across Asia, with more than 74 species presented. The most frequently documented taxa were *C. parvum* (*n* = 93), *C. andersoni* (*n* = 55), *C. bovis* (*n* = 43), and *C. ryanae* (*n* = 40). Among these, *C. parvum* demonstrated the broadest host range, being detected in more than 37 animal species, including livestock (cattle, calves, goats, sheep, camels, yaks), companion animals (dogs, cats, horses), wildlife (bats, deer, squirrels, apes, raccoons), and avian hosts. Similarly, *C. andersoni* showed broad host adaptability, predominating in cattle, calves, yaks, and camels, but also extending to non-traditional hosts such as macaques, pandas, swans, and bats.

By contrast, *C. bovis* and *C. ryanae* were primarily restricted to ruminants, particularly calves, cattle, and yaks, emphasizing their role in bovine cryptosporidiosis. Zoonotic species, including *C. hominis* (*n* = 14) and *C. meleagridis* (*n* = 13), were identified in multiple animal hosts, suggesting their potential role in cross-species transmission. In addition, several host-specific or geographically restricted genotypes (e.g., those of bats, chipmunks, rats, voles, and bamboo rats) were documented, suggesting ongoing host adaptation and the emergence of potential novel lineages.

Although less frequent, mixed infections were observed in cattle and goats, reflecting the co-circulation of multiple *Cryptosporidium* species within the same host populations. Collectively, these findings highlight the extensive species diversity and wide ecological host range of *Cryptosporidium* in Asia.

#### Distribution frequencies of *Cryptosporidium GP60* subtypes in animals across Asia

The subtyping analysis (Table 1, Additional file 9) revealed considerable heterogeneity in the distribution of *Cryptosporidium GP60* families across animal hosts in Asia. *C. parvum* showed the most extraordinary subtype diversity, with subtype family IId (*n* = 49) being the most prevalent, identified in more than 22 host species, including cattle, calves, goats, sheep, yaks, pigs, cats, dogs, horses, macaques, golden takins, pigeons, crows, and alpacas. Subtype family IIa (*n* = 13) was also common, particularly in calves, small ruminants, and rodents. In contrast, subtypes IIo (*n* = 11) and IIp (*n* = 4) occurred less frequently but were detected mainly in monkeys and rats.

**Table 1:** **Frequencies of *Cryptosporidium GP60* subtype families in animals across Asia.**

*Cryptosporidium* species	*Cryptosporidium* subtype family	The quantity of documented subtype families per study	The quantity of documented species per animal host		
			Total count	Number of hosts	Host name (s) (species quantity/host)
*C. bovis*	XXVIa	2	2	2	Calves (1), Cattle (1)
	XXVIb	2	2	2	Calves (1), Cattle (1)
	XXVIc	2	2	2	Calves (1), Cattle (1)
	XXVId	2	2	2	Calves (1), Cattle (1)
	XXVIe	2	2	2	Calves (1), Cattle (1)
	XXVIf	2	2	2	Calves (1), Cattle (1)
*C. canis*	XXa	4	4	2	Dog (3), Fox (1)
	XXb	1	1	1	Dogs (1)
	XXc	1	1	1	Dogs (1)
	XXd	1	1	1	Minks (1)
	XXe	1	1	1	Dogs (1)
	XXf	1	1	1	Minks (1)
	XXg	1	1	1	Fox (1)
*C. cuniculus*	Vb	1	1	1	Rabbits (1)
*C. erinacei*	XIIIb	1	1	1	Hedgehogs (1)
*C. felis*	XIXa	2	2	1	Cats (2)
	XIXb	1	1	1	Cats (1)
	XIXc	1	1	1	Cats (1)
	XIXd	1	1	1	Cats (1)
	XIXe	1	1	1	Cats (1)
*C. hominis*	Ia	1	1	1	Long-tailed macaques (1)
	Ib	3	3	3	Captive mammals (1), Dugongs (1), Nonhuman primates (1)
	Id	1	1	1	Horses (1)
	If	1	1	1	Nonhuman primates (1)
	Ii	3	3	3	Long-tailed macaques (1), Monkeys (1), Nonhuman primates (1)
	Ik	3	3	2	Camels (1), Donkeys (2)
	Im	1	1	1	Monkeys (1),
	In	1	1	1	Monkeys (1),
	Io	1	1	1	Long-tailed macaques (1)
*C. parvum*	IIo	2	2	2	Monkeys (1), Rats (1)
	IIa	13	16	9	**Calves (6),** Cattle (1), Deer (1), Dogs (1), Goats (2), Hens (1), Mouse (1), Rats (1), Sheep (2)
	IId	**49**	**52**	22	Alpacas (1), **Calves (13),** Camels (2), Cats (1), Cattle (7), Crows (1), Deer (3), Donkeys (2), Goats (2), Golden takins (1), Horses (1), Monkeys (1), Macaca mulatta (2), Minks (1), Nonhuman primates (1), Pigs (1), Pigeons (1), Rats (5), Sheep (3), shrew (1), Wild animals (1), Yaks (1)
	IIp	4	4	2	Nonhuman primates (1), **Rats (3)**
	If-like	2	2	1	Camels (2)
*C. tyzzeri*	IXa	1	1	1	Mouse (1)
	IXb	1	1	1	Mouse (1)
*C. meleagridis*	IIIb	5	5	1	Chicken (5)
	IIIe	3	3	3	Chicken (1), Different birds (1), Fox (1)
	IIIg	1	1	1	Chicken (1)
*C. ubiquitum*	XIIa	**14**	**18**	6	Camels (1), Chinchilla (1), Deer (4), Goats (1), **Sheep (10),** Yaks (1)
	XIId	3	3	2	**Chinchilla (2),** Monkeys (1)
	XIIh	2	2	1	Squirrel (2)
	XIIj	1	1	1	Mice (1)
	XIIb	1	1	1	Mice (1)
	XIIi	1	1	1	Squirrel (1)
*C. viatorum*	XVa	1	2	1	Rats (2)
	XVc	2	3	1	Rats (3)
	XVd	3	3	2	**Rats (2),** Shrew (1)
	XVe	1	2	2	Mice (1), Rats (1)
	XVf	1	2	2	Mice (1), Rats (1)
*C. xiaoi*	XXIIIa	2	2	2	Deer (1), Goat (1)
	XXIIIg	3	4	3	Deer (1), **Goats (2),** Sheep (1)
	XXIIIc	2	3	2	Goats (1), **Sheep (2)**
	XXIIId	2	2	2	Sheep (2)
	XXIIIe	1	1	1	Sheep (1)
	XXIIII	2	2	2	Sheep (2)
	XXIIIf	1	1	1	Goats (1)
	XXIIIa + XXIIIg	1	1	1	Goats (1)
*C. wrairi*	VIIa	1	2	1	Pigs (2)
*C. ryanae*	XXIa	1	1	1	Cattle (1)
	XXIb	1	1	1	Cattle (1)
	XXIf	1	1	1	Calves (1)
	XXId	1	1	1	Calves (1)
	XXIe	1	1	1	Calves (1)
	XXIg	1	1	1	Calves (1)
Horse genotype	VIa	3	4	3	Donkeys (2), Horses (2)
	VIb	1	1	1	Hedgehogs (1)
Mink genotype	Xc	1	1	1	Minks (1)
	Xe	1	1	1	Minks (1)
	Xf	1	1	1	Minks (1)
	Xg	1	1	1	Minks (1)
Skunk genotype	XVIa	1	2	1	Procyon lotor (2)

Bold numbers were the most frequent genotype/subtype per source. Host names were arranged in alphabetical order. The highest frequencies of hosts are in bold text

*C.: Cryptosporidium*

*C. ubiquitum* was dominated by subtype XIIa (*n* = 14), affecting sheep, goats, camels, chinchillas, deer, and yaks, whereas XIIh (*n* = 11) was unique to squirrels, indicating strong host specificity. *C. hominis* exhibited moderate subtype diversity, with Ib (*n* = 3), Ii (*n* = 3), and Ik (*n* = 3) detected in non-human primates, camels, donkeys, and dugongs, suggesting occasional cross-species transmission. *C. meleagridis* was represented by IIIb, IIIe, and IIIg, mainly in poultry and wild birds. Similarly, *C. canis* subtypes (XXa–g) were recorded in dogs, foxes, and minks, while *C. xiaoi* subtypes (XXIIIa, c–g, I) were mainly confined to sheep and goats.

Several rare or host-adapted subtype families were also observed, such as *C. bovis* (XXVIa–f), *C. felis* (XIXa–e), and *C. viatorum* (XVa, c–f), reflecting the broad genetic spectrum of animal-associated *Cryptosporidium* across the continent. Collectively, the predominance of zoonotic subtypes, particularly *C. parvum* IId and *C. ubiquitum* XIIa, emphasizes their epidemiological importance and role in transmission dynamics at the human-animal-environment interface across Asia.

#### Distribution frequencies of *Cryptosporidium* MLST–subtypes in animals across Asia

Multi-locus sequence typing (MLST) analysis (Table 2, Additional file 9) of *C. andersoni* revealed limited but distinct subtype diversity across livestock and wildlife hosts in Asia. The predominant subtype combination A4, A4, A4, A1 was consistently identified in Holstein calves, dairy cattle, and golden takins, indicating its widespread circulation among both domestic and wild ruminants. Other subtype profiles, including A5, A4, A2, A1, and A1, A4, A2, A1, were reported in yaks and farm cattle, while additional allelic combinations such as A4, A5, A2, A1, and A4, A5, A4, A1 were also detected in cattle populations. These findings suggest both clonal expansion of dominant subtype families and localized genetic variability shaped by host species and geography. Overall, the detection of multiple MLST-defined subtypes of *C. andersoni* across calves, yaks, cattle, and golden takins underscores the adaptability of these species in ruminants and highlights the value of MLST in resolving intra-species diversity relevant to transmission dynamics in Asia.

**Table 2:** **Frequencies of *Cryptosporidium* multi-locus sequence typing subtypes families in animals across Asia.**

*Cryptosporidium* species	*Cryptosporidium* subtype	Microsatellites used	Studies no.	Animal host
*C. andersoni*	A4, A4, A4, A1	MS1, MS2, MS3, MS16	1	Holstein calves
	A5, A4, A2, A1		1	Yaks
	A1, A4, A2, A1 A4, A5, A2, A1 A4, A5, A4, A1		1	Farm cattle
	A1, A4, A4, A1		1	Goldin takins
	A4, A4, A4, A1 A2, A5, A2, A1		1	Dairy cattle

## Discussion

The current review highlights both the breadth and the critical gaps in research on *Cryptosporidium* in animals across Asia over the past decade, with studies spanning 29 of the 50 countries, leaving substantial geographic blind spots in Central, Western, and Southeast Asia. Such uneven representation reflects broader disparities in regional research capacity, surveillance infrastructure, and prioritization of zoonotic diseases [4, 25, 33]. The dominance of livestock-focused studies (*n* = 254) is consistent with global concerns about the agricultural impact and zoonotic potential of *Cryptosporidium* [34, 35], yet the relatively limited attention to wildlife, stray, and captive populations is concerning, given their recognized role in parasite transmission at the human-animal-environment interface [36]. Moreover, the frequent omission of basic demographic data, such as 108 studies lacking age information and 193 failing to report clinical status, represents a significant limitation that hinders epidemiological interpretation and risk assessment. Previous work has emphasized that host age and clinical presentation are key determinants of infection dynamics, with young and immunocompromised animals typically exhibiting higher susceptibility and more severe outcomes [37, 38]. The spectrum of clinical signs recorded in symptomatic cases, ranging from diarrhea and anorexia to neurological impairment and mortality, further underscores the multifaceted impact of cryptosporidiosis across taxa, consistent with observations in the veterinary contexts [39]. These findings reveal that while recent years have seen notable advances in the surveillance of *Cryptosporidium* in Asia, significant gaps persist in geographic coverage, host range, and clinical characterization.

The pooled prevalence of *Cryptosporidium* spp. across 329 studies, estimated at 12%, underscores the considerable burden of infection among diverse animal hosts just in the past decade across Asia and is broadly consistent with global meta-analyses reporting prevalence rates ranging from 9 to 15% depending on host taxa and geographical context [4, 34, 40], where animal infections have been shown to play a key role in sustaining environmental contamination and cross-species transmission. In Asia, the high density of livestock farming, close human-animal contact, and widespread reliance on untreated surface water for agricultural and domestic use are likely to exacerbate exposure risks [41, 42]. The prevalence records reinforce the importance of harmonized surveillance frameworks and standardized detection/diagnostic approaches better to capture the epidemiological landscape of *Cryptosporidium* in animal populations.

The regional stratification of Asia revealed a distinct geographical gradient in *Cryptosporidium* prevalence, with the highest pooled estimate observed in West Asia, followed by Southeast Asia, South Asia, East Asia, and North Asia. At the same time Central Asia was notably absent from the available literature. Such variation likely reflects complex interactions between climatic conditions, livestock density, husbandry practices, surveillance capacity across regions, and possibly, research interest. Arid and semi-arid environments characteristic of many West Asian countries may facilitate water scarcity and contamination of limited shared water resources, thereby enhancing parasite transmission among animals and humans [33, 43]. In contrast, the comparatively lower prevalence in North Asia may be attributed to its colder climate, which is less conducive to oocyst survival and transmission, as supported by experimental studies demonstrating reduced viability of *Cryptosporidium* oocysts under freezing conditions [44]. Regional differences in animal production systems also play a pivotal role, as intensive farming in South and Southeast Asia often increases exposure risk. Additionally, disparities in diagnostic approaches, study design, and research prioritization contribute to observed heterogeneity, with countries in West and South Asia showing greater surveillance of livestock and zoonotic reservoirs compared to underrepresented regions such as Central Asia [4, 39]. These findings emphasize that the prevalence of *Cryptosporidium* in Asia is shaped not only by biological and ecological determinants but also by anthropogenic factors [25].

The temporal analysis of studies on *Cryptosporidium* in Asian animals between 2015 and 2024 revealed no consistent trend in study numbers, sample sizes, or reported prevalence, even when considering partial data from 2025. Notably, the sharp increase in sample sizes in 2019 and 2024 does not appear linked to any identifiable drivers within the scope of this review, such as disease outbreaks or methodological shifts. Furthermore, fluctuations in sample size did not correspond to changes in prevalence estimates, suggesting that the parasite burden may be relatively stable across time and host populations. This observation could indicate a broadly uniform distribution of *Cryptosporidium* in the region, possibly reflecting the diverse stratification of host species under investigation, which is consistent with previous reports highlighting the parasite's wide ecological adaptability and host range [4, 38].

The cross-kingdom comparison demonstrated that *Cryptosporidium* spp. was detected in both vertebrates and invertebrates. While vertebrates accounted for most samples with a pooled prevalence of 10.8%, invertebrates showed a higher prevalence of 13.9% across six studies. Invertebrates, such as mollusks and arthropods, may not be actual biological hosts but act as environmental reservoirs or mechanical vectors, often accumulating oocysts through filter-feeding or contact with contaminated substrates [45, 46]. Consequently, their prevalence estimates may be artificially elevated, reflecting environmental contamination levels rather than active infections. Similarly, the variation in *Cryptosporidium* prevalence across the seven animal classes identified adds to the previous concept. The markedly high test-positive rates in mollusks (22.9%) and poriferans (20%) support the hypothesis that filter-feeding aquatic invertebrates bioaccumulate oocysts from contaminated environments, thereby functioning as environmental sentinels and amplifiers of waterborne transmission [47, 48]. These findings collectively support the hypothesis that *Cryptosporidium* transmission is powerfully shaped by ecological niche and host physiology, with aquatic filter feeders serving as reservoirs and dispersal agents. In contrast, vertebrate data provides a more direct measure of infection dynamics within host populations.

The finding that livestock represented both the most extensively investigated host group and the category with the highest pooled prevalence of *Cryptosporidium* spp. (13.2%) supports the hypothesis that these animals constitute a central reservoir in the parasite’s transmission cycle. A plausible explanation lies in the biology of young ruminants, particularly calves, which are highly susceptible to infection and shed vast numbers of oocysts into the environment, thereby amplifying contamination pressure on shared water sources and pastures [49, 50]. This pattern suggests that livestock, especially neonatal cohorts, may function as epidemiological “super-shedders,” driving sustained transmission within herds and facilitating spillover to humans and other animals. Furthermore, the persistence of environmentally resistant oocysts, combined with intensive farming practices and close animal contact, may explain why livestock exhibit a higher prevalence than other animal classes [4, 51]. Taken together, these results suggest that targeted control measures in calves and other young livestock could disproportionately reduce both agricultural losses and zoonotic transmission risk.

The mismatch observed in this review between the most frequently sampled animals and those harboring the highest prevalence of *Cryptosporidium* carries significant implications for emerging risks and zoonotic spillover. While primates, dogs, and livestock were the focus of sampling, species such as lynxes, lions, sugar gliders, raccoon dogs, bivalves, crows, and dugongs exhibited higher infection burdens, highlighting overlooked pathways of transmission. This pattern raises the hypothesis that unconventional hosts may contribute disproportionately to public health risks despite their limited representation in surveillance datasets. Aquatic organisms such as bivalves and dugongs, for example, may act as bio-accumulators of oocysts, thereby posing foodborne and waterborne threats when consumed or through contamination of coastal ecosystems [52, 53]. Similarly, the high prevalence in scavenging species such as crows and rodents underscore their potential role as amplifiers at the human–environment interface, particularly in urban areas where they thrive on waste and contaminated resources [15, 54]. The elevated infection rate in exotic or less conventional pets or animals used for commercial purposes, such as sugar gliders and raccoon dogs, further underscores the zoonotic risk associated with the growing trade and the close contact of humans with novel companions or species for commercial use [55, 56]. Such findings suggest that *Cryptosporidium* transmission risks are not confined to traditional livestock or domestic species but extend to a diverse array of hosts, many of which intersect with human food systems/fur trade/animal research, urban environments, and wildlife trade.

Across Asia, the distribution of *Cryptosporidium* species in animals illustrates distinct evolutionary and ecological strategies that underpin their transmission dynamics. The remarkable adaptability of *C. parvum* across a wide array of hosts suggests that this species possesses traits such as flexibility in host-cell invasion and immune evasion, that enable successful colonization of taxonomically diverse animals. These features make *C. parvum* a principal driver of zoonotic cryptosporidiosis in the region [4, 57]. In contrast, the detection of *C. andersoni* in unconventional hosts, including macaques, pandas, swans, and bats, suggests ecological overlaps at shared water sources or feeding habitats as a likely mechanism for host switching. Such findings imply that *C. andersoni*, traditionally regarded as a ruminant-adapted parasite, may be expanding its ecological niche in response to changing agricultural and environmental pressures [4, 58, 59]. By comparison, *C. bovis* and *C. ryanae* exhibit a narrower host spectrum, primarily confined to ruminants, including calves, cattle, and yaks. Their distribution emphasizes their role as age-associated pathogens of bovine cryptosporidiosis, reflecting specialization shaped by host physiology and immune immaturity in young animals. While this limits their zoonotic potential, these species nonetheless contribute to maintaining endemic diseases within livestock systems [60].

Other zoonotic taxa, including *C. hominis* and *C. meleagridis*, were also identified in multiple animal species, underscoring their cross-species transmission potential and highlighting Asia as a key region where human and animal *Cryptosporidium* biodiversity overlap [25, 41, 61]. Furthermore, the detection of several host-specific or geographically restricted genotypes, such as those from bats, chipmunks, rats, voles, and bamboo rats, indicates ongoing host adaptation and the possible emergence of novel evolutionary lineages. These observations suggest that wildlife reservoirs play a crucial role in maintaining genetic diversity and have the potential to generate new variants that can infect humans under shifting ecological or anthropogenic conditions. Notably, the occurrence of mixed-species infections, sometimes involving up to three *Cryptosporidium* species within a single host, raises the prospect of genetic recombination and accelerated parasite evolution. Such dynamics are likely intensified in mixed-host farming systems or at wildlife-livestock interfaces, which may act as hotspots for the emergence of novel genotypes with heightened transmissibility or virulence [36].

Building on the remarkable species diversity of *Cryptosporidium* documented across Asia, the distribution of *gp60* subtype families revealed striking heterogeneity, with *C. parvum* emerging as the most genetically diverse taxon, dominated by the IId family but also encompassing a wide spectrum of subtype variants. The broad representation of IId across more than 22 host species suggests that this lineage has evolved adaptive traits enabling persistence across multiple host ecologies, consistent with its frequent detection in both livestock and wildlife in Asia [57, 60]. By contrast, subtypes such as XIIh in squirrels or XXIIIa-l in small ruminants illustrate host-restricted patterns that likely reflect long-term coevolutionary stability within specialized ecological niches [62]. Rare or geographically restricted subtypes, such as those of *C. bovis*, *C. felis*, and *C. viatorum*, further underscore the hidden reservoirs of genetic diversity circulating within Asian animal populations.

Taken together, the previous evidence suggests that Asia is not only a hotspot of *Cryptosporidium* prevalence but also a dynamic arena of host-parasite interactions, where generalist, specialist, and potentially emerging lineages coexist. Future research should prioritize addressing the geographic and host-related gaps identified in this review. Expanded and systematic surveillance in Central, Western, and Southeast Asian countries, particularly in settings with limited diagnostic and laboratory infrastructure, is crucial for accurately delineating the regional distribution of *Cryptosporidium* and detecting potentially novel species or subtypes. In parallel, broadening investigative efforts beyond conventional livestock to encompass wildlife, free-ranging animals, and captive exotic species will be critical for elucidating transmission dynamics at the human-animal interface. Longitudinal study designs integrating demographic, clinical, and molecular data are further warranted to assess age-related susceptibility, infection outcomes, and disease severity across diverse host populations. Consistent documentation of age, clinical status, and sampling frameworks would substantially improve cross-study comparability and mitigate systematic bias. Ultimately, the implementation of integrated One Health frameworks emphasizes the interconnectedness of human, animal, and environmental health in anticipating and minimizing zoonotic risk. Operationalizing such frameworks necessitates coordinated, multisectoral strategies, including the establishment of cross-sectoral surveillance systems that integrate human, veterinary, and environmental monitoring; the development and harmonization of standardized diagnostic protocols across diverse host taxa; and the advancement of translational interventions, such as vaccines and environmental management strategies, targeting both livestock and vulnerable human populations. By situating the findings of this meta-analysis within a One Health paradigm, these approaches provide a strategic roadmap for evidence-informed, preemptive measures to reduce the burden of cryptosporidiosis across Asia and beyond.

The authors acknowledge several limitations of this review. First, the literature coverage was restricted to studies published in English. Consequently, although China contributed a substantial proportion of the included studies compared with other Asian countries, additional relevant literature indexed in Chinese-language databases (e.g., CNKI and the Chinese Dissertations Database via Wanfang) may not have been captured, primarily due to practical constraints related to language accessibility and translation. Second, there was a possibility of publication bias, noting that studies reporting null or low prevalence estimates may be underrepresented in the published literature. Third, several studies that met the inclusion criteria could not be retrieved due to restricted institutional access or paywall barriers, underscoring broader concerns about inequitable access to scientific knowledge under prevailing publication models. Fourth, a pronounced imbalance was observed in the number of studies focusing on invertebrate versus vertebrate hosts, which may partly account for the higher reported prevalence estimates in invertebrates. This uneven distribution of studies and sampled animals may limit the statistical robustness of the prevalence estimates and highlights a notable research gap in *Cryptosporidium* investigations involving invertebrate hosts.

## Conclusion

This systematic review has established an evidence-based narrative of the expanding threat of *Cryptosporidium* infections among animals across Asia from 2015 to 2025. The overall pooled prevalence was estimated at 12%, and the molecular analysis revealed the circulation of at least seven distinct *Cryptosporidium* species with recognized zoonotic relevance, among which *C. parvum* was most frequently reported. Spatially, higher prevalence was observed in parts of West Asia and Southeast Asia, regions characterized by intensive livestock production and, in some settings, limited regulation of animal movement. Collectively, these findings indicate that *Cryptosporidium* spp. represent a persistent and regionally variable parasitic burden rather than an isolated veterinary issue. The observed heterogeneity across host groups and geographic areas highlights the need for enhanced diagnostic coverage, standardized molecular surveillance, and stronger collaboration between veterinary, public health, and environmental sectors. Strengthening such integrated approaches may support more accurate risk assessment and inform strategies to reduce transmission at the animal-human–environment interface in Asia.

## Supplementary Information

**Additional file 1: PRISMA checklist.**

**Additional file 2: Search strategy of the MeSH Keywords and the MeSH terms.**

**Additional file 3: PRISMA flow diagram of literature search and included studies.**

**Additional file 4: Meta-analysis tables and figures (Excel file).**

**Additional file 5: Overall proportional prevalence of *Cryptosporidium* spp. within the different pooled animals across the countries investigated.**

**Additional file 6: Funnel plot of overall prevalence proportion of *Cryptosporidium* spp. within the pooled animals assessing publication bias across the countries investigated.**

**Additional file 7: Perspectives on the actual relationships among lowest and highest “countries, prevalence rates, sample sizes, and the number of studies”.**

**Additional file 8: Overall proportional prevalence of *Cryptosporidium* spp. within the different methods used for its detection in Asian animals.**

**Additional file 9:** ***Cryptosporidium* genotyping studies (Excel file).**

**Additional file 10: Frequencies of *Cryptosporidium* species in animals across Asia.**

## Data Availability

All relevant data are included in the paper or its Additional Files.
