# Supplementary material for: Cryptosporidium infections in animals across Asia (2015–2025): a systematic review and meta-analysis of prevalence, host range, geographic distribution, and molecular epidemiology
Source: Vet Res. 2026 Apr 28;57:57. doi: 10.1186/s13567-026-01722-0 (PMC13123031; doi:10.1186/s13567-026-01722-0)
Supplement: Supplementary file 2 — Additional file 2: Search strategy of the MeSH Keywords and the MeSH terms. [file 13567_2026_1722_MOESM2_ESM.docx]

Additional File 2: Search strategy of the MeSH Keywords and the MeSH terms.

| **Search time** | **Database** | **Item** | **Keyword** | **Mesh terms** | **Result** |
| --- | --- | --- | --- | --- | --- |
| January 1^st^, 2015  to  February 6^th^, 2025 | PubMed | *Cryptosporidium* / cryptosporidiosis | *Cryptosporidium* | "cryptosporidium"[MeSH Terms] OR "cryptosporidium"[All Fields] | - |
|  |  | Asian countries | Yemen | "yemen"[MeSH Terms] OR "yemen"[All Fields] | 3 |
|  |  |  | Vietnam | "vietnam"[MeSH Terms] OR "vietnam"[All Fields] OR "vietnam s"[All Fields] | 13 |
|  |  |  | Uzbekistan | "uzbekistan"[MeSH Terms] OR "uzbekistan"[All Fields] | 0 |
|  |  |  | UAE | "united arab emirates"[MeSH Terms] OR "united"[All Fields] AND "arab"[All Fields] AND "emirates"[All Fields]) OR "united arab emirates"[All Fields]) AND "UAE"[All Fields] | 6 |
|  |  |  | Turkmenistan | "turkmenistan"[MeSH Terms] OR "turkmenistan"[All Fields] | 0 |
|  |  |  | Turkey | "turkey"[MeSH Terms] OR "turkey"[All Fields] OR "turkey s"[All Fields] OR "turkeys"[MeSH Terms] OR "turkeys"[All Fields] | 65 |
|  |  |  | Timor-Leste | "timor leste"[MeSH Terms] OR "timor leste"[All Fields] OR "timor"[All Fields] AND "leste"[All Fields] OR "timor leste"[All Fields] | 1 |
|  |  |  | Thailand | "thailand"[MeSH Terms] OR "thailand"[All Fields] OR "thailand s"[All Fields] | 49 |
|  |  |  | Tajikistan | "tajikistan"[MeSH Terms] OR "tajikistan"[All Fields] |  |
|  |  |  | Taiwan | "taiwan"[MeSH Terms] OR "taiwan"[All Fields] OR "taiwan s"[All Fields] OR "taiwans"[All Fields] | 14 |
|  |  |  | Syria | "syria"[MeSH Terms] OR "syria"[All Fields] OR "syria s"[All Fields] | 3 |
|  |  |  | Sri Lanka | "sri lanka"[MeSH Terms] OR "sri"[All Fields] AND "lanka"[All Fields] OR "sri lanka"[All Fields] | 7 |
|  |  |  | South Korea | "republic of korea"[MeSH Terms] OR ("republic"[All Fields] AND "korea"[All Fields]) OR "republic of korea"[All Fields] OR ("south"[All Fields] AND "korea"[All Fields]) OR "south korea"[All Fields] | 44 |
|  |  |  | Singapore | "singapore"[MeSH Terms] OR "singapore"[All Fields] OR "singapore s"[All Fields] | 22 |
|  |  |  | Saudi Arabia | "saudi arabia"[MeSH Terms] OR ("saudi"[All Fields] AND "arabia"[All Fields]) OR "saudi arabia"[All Fields] | 65 |
|  |  |  | Russia | "russia"[MeSH Terms] OR "russia"[All Fields] OR "russia s"[All Fields] OR "russias"[All Fields] | 4 |
|  |  |  | Qatar | "qatar"[MeSH Terms] OR "qatar"[All Fields] OR "qatar s"[All Fields] | 7 |
|  |  |  | Philippine | "philippine"[All Fields] OR "philippines"[MeSH Terms] OR "philippines"[All Fields] | 22 |
|  |  |  | Palestine | "Palestine"[All Fields] | 3 |
|  |  |  | Pakistan | "pakistan"[MeSH Terms] OR "pakistan"[All Fields] OR "pakistan s"[All Fields] | 50 |
|  |  |  | Oman | "oman"[MeSH Terms] OR "oman"[All Fields] | 1 |
|  |  |  | North Korea | "north"[All Fields] OR "norths"[All Fields]) AND ("korea"[MeSH Terms] OR "korea"[All Fields] OR "korea s"[All Fields] OR "koreas"[All Fields] | 0 |
|  |  |  | Nepal | "nepal"[MeSH Terms] OR "nepal"[All Fields] OR "nepal s"[All Fields] | 26 |
|  |  |  | Myanmar | "myanmar"[MeSH Terms] OR "myanmar"[All Fields] OR "myanmar s"[All Fields] OR "myanmars"[All Fields] | 5 |
|  |  |  | Mongolia | "mongolia"[MeSH Terms] OR "mongolia"[All Fields] OR "mongolia s"[All Fields] | 14 |
|  |  |  | Maldives | "maldive"[All Fields] OR "maldives"[MeSH Terms] OR "maldives"[All Fields] | 0 |
|  |  |  | Malaysia | "malaysia"[MeSH Terms] OR "malaysia"[All Fields] OR "malaysia s"[All Fields] | 32 |
|  |  |  | Lebanon | "lebanon"[MeSH Terms] OR "lebanon"[All Fields] OR "lebanon s"[All Fields] | 14 |
|  |  |  | Laos | "laos"[MeSH Terms] OR "laos"[All Fields] | 0 |
|  |  |  | Kyrgyzstan | "kyrgyzstan"[MeSH Terms] OR "kyrgyzstan"[All Fields] | 0 |
|  |  |  | Kuwait | "kuwait"[MeSH Terms] OR "kuwait"[All Fields] OR "kuwait s"[All Fields] | 7 |
|  |  |  | Kazakhstan | "kazakhstan"[MeSH Terms] OR "kazakhstan"[All Fields] OR "kazakhstan s"[All Fields] | 3 |
|  |  |  | Jordan | "jordan"[MeSH Terms] OR "jordan"[All Fields] | 21 |
|  |  |  | Japan | "japan"[MeSH Terms] OR "japan"[All Fields] OR "japan s"[All Fields] OR "japans"[All Fields] | 89 |
|  |  |  | Israel | "israel"[MeSH Terms] OR "israel"[All Fields] OR "israel s"[All Fields] | 24 |
|  |  |  | Iraq | "iraq"[MeSH Terms] OR "iraq"[All Fields] | 18 |
|  |  |  | Iran | "iran"[MeSH Terms] OR "iran"[All Fields] | 157 |
|  |  |  | Indonesia | "indonesia"[MeSH Terms] OR "indonesia"[All Fields] OR "indonesia s"[All Fields] OR "indonesias"[All Fields] | 28 |
|  |  |  | India | "india"[MeSH Terms] OR "india"[All Fields] OR "india s"[All Fields] OR "indias"[All Fields] | 169 |
|  |  |  | Georgia | "georgia"[MeSH Terms] OR "georgia"[All Fields] OR "georgia republic"[MeSH Terms] OR ("georgia"[All Fields] AND "republic"[All Fields]) OR "georgia republic"[All Fields] OR "georgia s"[All Fields] | 110 |
|  |  |  | Cyprus | "cyprus"[MeSH Terms] OR "cyprus"[All Fields] | 36 |
|  |  |  | Cambodia | "cambodia"[MeSH Terms] OR "cambodia"[All Fields] OR "cambodia s"[All Fields] | 5 |
|  |  |  | Brunei | "brunei"[MeSH Terms] OR "brunei"[All Fields] | 0 |
|  |  |  | Bhutan | "bhutan"[MeSH Terms] OR "bhutan"[All Fields] OR "bhutan s"[All Fields] | 0 |
|  |  |  | Bangladesh | "bangladesh"[MeSH Terms] OR "bangladesh"[All Fields] OR "bangladesh s"[All Fields] | 78 |
|  |  |  | Bahrain | "bahrain"[MeSH Terms] OR "bahrain"[All Fields] | 0 |
|  |  |  | Azerbaijan | "azerbaijan"[MeSH Terms] OR "azerbaijan"[All Fields] | 5 |
|  |  |  | Armenia | "armenia"[MeSH Terms] OR "armenia"[All Fields] | 5 |
|  |  |  | China | "china"[MeSH Terms] OR "china"[All Fields] OR "china s"[All Fields] OR "chinas"[All Fields] | 611 |
|  |  |  | Afghanistan | "afghanistan"[MeSH Terms] OR "afghanistan"[All Fields] OR "afghanistan s"[All Fields] | 2 |
|  |  | Filter | 10 years | y_10[Filter] | 1838 |
