# Supplementary material for: Cryptosporidium infections in animals across Asia (2015–2025): a systematic review and meta-analysis of prevalence, host range, geographic distribution, and molecular epidemiology
Source: Vet Res. 2026 Apr 28;57:57. doi: 10.1186/s13567-026-01722-0 (PMC13123031; doi:10.1186/s13567-026-01722-0)
Supplement: Supplementary file 3 — Additional file 3: PRISMA flow diagram of literature search and included studies. [file 13567_2026_1722_MOESM3_ESM.docx]

Records removed before screening:

Duplicate records removed

(n = 339)

Records identified from:

Pubmed (n = 1838)

**Identification**

Records excluded (n = 1058)

Not related to the study and findings of interest (n = 1058)

Records screened for title and abstract (n = 1499)

Reports not retrieved (n = 43).

No full-text available or required paid access.

Reports sought for retrieval (n = 441)

**Screening**

Reports excluded (n = 85):

Unrelated to objectives (n = 68)

No prevalence reported (n = 13)

Articles with ambiguous data (n = 4).

Reports assessed for full-text eligibility (n = 398)

Studies retrieved from the reference list (n = 16)

Studies included from PubMed (n 313)

Studies included in the systematic review and meta-analysis (n = 329)

**Included**

**Additional File 3:** PRISMA flow diagram of literature search and included studies. PRISMA flow diagram.
