# Supplementary material for: Cryptosporidium infections in animals across Asia (2015–2025): a systematic review and meta-analysis of prevalence, host range, geographic distribution, and molecular epidemiology
Source: Vet Res. 2026 Apr 28;57:57. doi: 10.1186/s13567-026-01722-0 (PMC13123031; doi:10.1186/s13567-026-01722-0)
Supplement: Supplementary file 4 — Additional file 4: Meta-analysis tables and figures (Excel file). [file 13567_2026_1722_MOESM4_ESM.docx]

**Additional File 4:** Overall proportional prevalence of *Cryptosporidium* spp. within the different pooled animals across the countries investigated.
