# Supplementary material for: Cryptosporidium infections in animals across Asia (2015–2025): a systematic review and meta-analysis of prevalence, host range, geographic distribution, and molecular epidemiology
Source: Vet Res. 2026 Apr 28;57:57. doi: 10.1186/s13567-026-01722-0 (PMC13123031; doi:10.1186/s13567-026-01722-0)

**Additional File 5:** Funnel plot of overall prevalence proportion of *Cryptosporidium* spp. within the pooled animals assessing publication bias across the countries investigated.


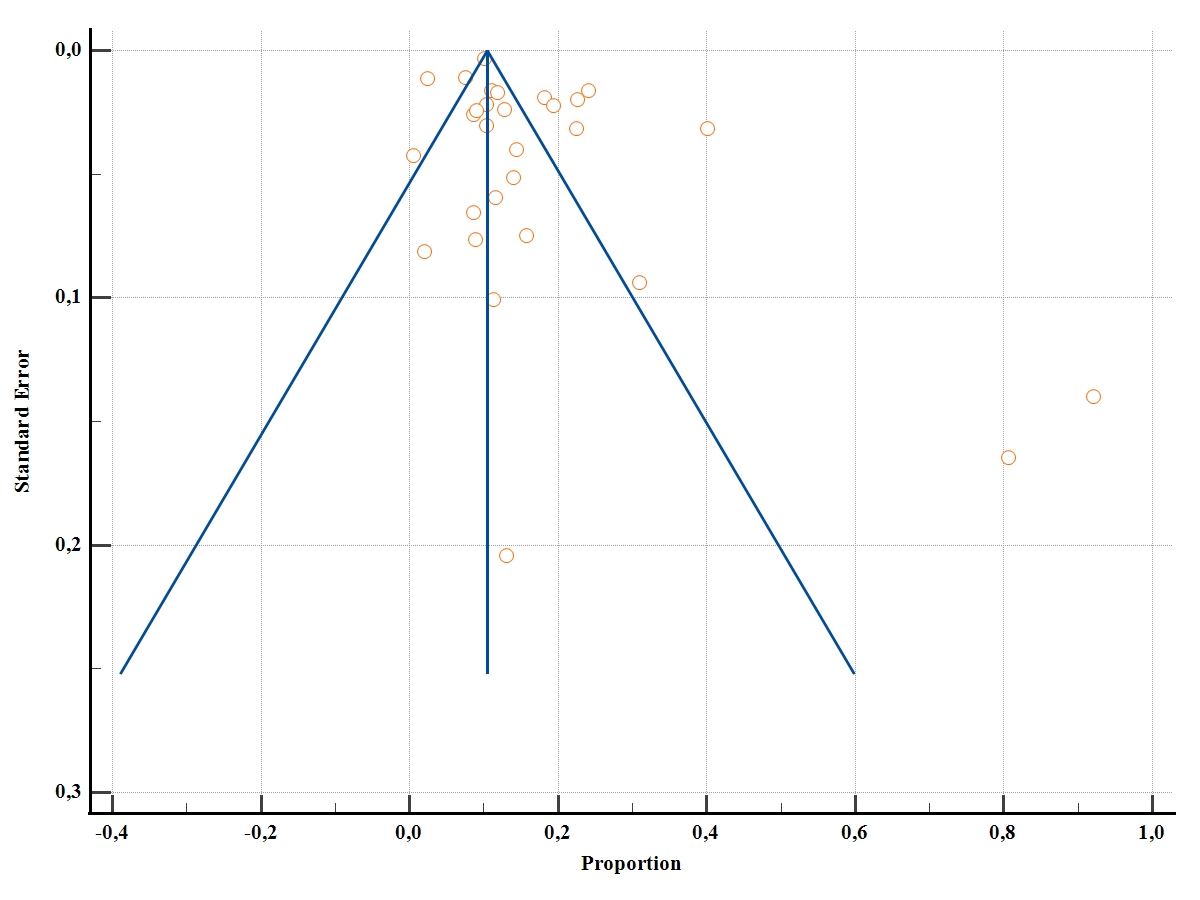

Supplement: Supplementary file 5 — Additional file 5: Overall proportional prevalence of Cryptosporidium spp. within the different pooled animals across the countries investigated. [file 13567_2026_1722_MOESM5_ESM.docx]
