# Supplementary material for: Cryptosporidium infections in animals across Asia (2015–2025): a systematic review and meta-analysis of prevalence, host range, geographic distribution, and molecular epidemiology
Source: Vet Res. 2026 Apr 28;57:57. doi: 10.1186/s13567-026-01722-0 (PMC13123031; doi:10.1186/s13567-026-01722-0)
Supplement: Supplementary file 6 — Additional file 6: Funnel plot of overall prevalence proportion of Cryptosporidium spp. within the pooled animals assessing publication bias across the countries investigated. [file 13567_2026_1722_MOESM6_ESM.docx]

**Additional File 6:** Perspectives on the actual relationships among lowest and highest “countries, prevalence rates, sample sizes, and the number of studies”.

| **Item** | **Notes** | **Country** | **Prevalence** | **Sample size** | **No. of studies** | **Prevalence rank /Item** |
| --- | --- | --- | --- | --- | --- | --- |
| **Prevalence** | Highest | Israel | 92% | 50 | 1 | 1^st^ |
|  | Lowest | Mongolia | 0.5% | 555 | 1 | 29^th^ |
| **Sample size** | Highest | China | 10.1% | 94,945 | 180 | 21^st^ |
|  | Lowest | Qatar | 13.0% | 23 | 2 | 13^th^ |
| **Studies No.** | Highest | China | 10.1% | 94,945 | 180 | 21^st^ |
|  | Lowest | Mongolia | 0.5% | 555 | 1 | 29^th^ |
|  |  | Palestine | 2% | 150 | 1 | 28^th^ |
|  |  | Azerbaijan | 8.8% | 170 | 1 | 23^rd^ |
|  |  | Sri Lanka | 11.2% | 98 | 1 | 17^th^ |
|  |  | Jordan | 11.6% | 284 | 1 | 16^th^ |
|  |  | Syria | 15.7% | 178 | 1 | 10^th^ |
|  |  | Vietnam | 31% | 113 | 1 | 4^th^ |
|  |  | Saudi Arabia | 40% | 36 | 1 | 3^rd^ |
|  |  | UAE | 80.6% | 24 | 1 | 2^nd^ |
|  |  | Israel | 92% | 50 | 1 | 1^st^ |
