# Supplementary material for: Cryptosporidium infections in animals across Asia (2015–2025): a systematic review and meta-analysis of prevalence, host range, geographic distribution, and molecular epidemiology
Source: Vet Res. 2026 Apr 28;57:57. doi: 10.1186/s13567-026-01722-0 (PMC13123031; doi:10.1186/s13567-026-01722-0)
Supplement: Supplementary file 8 — Additional file 8: Overall proportional prevalence of Cryptosporidium spp. within the different methods used for its detection in Asian animals. [file 13567_2026_1722_MOESM8_ESM.docx]

**Additional File 8:** Frequencies of *Cryptosporidium* species in animals across Asia.

| **Serial** | ***Cryptosporidium* species/genotypes*** | **The quantity of documented species/genotypes per Asian studies** | **The quantity of documented species per animal host** | | |
| --- | --- | --- | --- | --- | --- |
|  |  |  | **Total count** | **Number of hosts** | **Host name (s) (species quantity/host)** |
| 1 | *C. alticolis* | 1 | 1 | 1 | Voles (1) |
| 2 | *C. andersoni* | **55** | **68** | 20 | Artiodactyla species (1), Bats (2), Birds (2), Calves (12), Camels (3), Captive mammals (1), **Cattle (21)**, Dairy (2), Deer (1), Donkeys (1), Goats (2), Hamster (3), Horses (1), Macaques (2), Swan (1), Sheep (3), Panda (3), Takins (1), Wild Animals (1), Yaks (5) |
| 3 | *C. avium* | 1 | 1 | 1 | Birds (1) |
| 4 | *C. avian* genotype III | 3 | 4 | 3 | Migratory ducks (1), **Peafowl (2),** Psittaciformes (1) |
| 5 | *C. avian* genotype V | 2 | 2 | 2 | Birds (1), Wild animals (1) |
| 6 | *C. baileyi* | 20 | 23 | 9 | Birds (5), **Chicken (8)**, Ducks (3), Galliformes (1), Geese (2), Goats (1), Psittaciformes (1), Rats (1), Sparrows (1) |
| 7 | *C.* bamboo rat genotype II | 2 | 2 | 2 | Bamboo rats (Rhizomys sinensis) (1), Masked palm civets (1) |
| 8 | *C.* bat genotype II | 2 | 3 | 1 | Bats (3) |
| 9 | *C.* bat genotype V | 1 | 1 | 1 | Bats (1) |
| 10 | *C.* bat genotype VI | 2 | 3 | 1 | Bats (1) |
| 11 | *C.* bat genotype VII | 1 | 1 | 1 | Bats (1) |
| 12 | *C.* bat genotype XXI | 1 | 1 | 1 | Bats (1) |
| 13 | *C.* bat genotype XXII | 1 | 1 | 1 | Bats (1) |
| 14 | *C. bear* genotype | 1 | 1 | 1 | Field mice (1) |
| 15 | *C. bovis* | **43** | **52** | 10 | Buffaloes (1), Calves (22), Camels (2), **Cattle (16),** Dairy (1), Deer (1), Donkeys (1), Goats (1), Sheep (1), Yaks (6) |
| 16 | *C. canis* | 23 | 31 | 8 | Calves (1), Cats (1), **Dogs (13)**, Foxes (6), Lambs (1), Minks (4), Racoon dogs (3), Voles (2) |
| 17 | *C. cervine* | 1 | 1 | 1 | Wild rodents and insectivore (1) |
| 18 | *C. chipmunk* genotype I | 1 | 1 | 1 | Wild rodents and insectivore (1) |
| 19 | *C. chipmunk* genotype III | 2 | 2 | 1 | Squirrel (2) |
| 20 | *C. chipmunk* genotype V | 3 | 4 | 4 | Chinchilla (1), Chipmunk (1), Marmot (1), Squirrel (1) |
| 21 | *C. cuniculus* | 1 | 1 | 1 | Rabbits (1) |
| 22 | *C.* deer genotype | 9 | 12 | 2 | Captive mammals (1), **Deer (11)** |
| 23 | *C. ditrichi* | 1 | 1 | 1 | Rats (1) |
| 24 | *C. erinacei* | 2 | 2 | 2 | Hedgehogs (1), Rats (1) |
| 25 | *C. felis* | 16 | 17 | 4 | Calves (1), Captive mammals (1), **Cats (14)**, Squirrel (1) |
| 26 | *C.* ferret genotype | 3 | 3 | 3 | Chipmunks (1), Squirrels (1), Rat (1) |
| 27 | *C. galli* | 6 | 7 | 6 | **Birds (3),** Chickens (1), Passeriformes (1), Psittaciformes (1), Wildlife animals (1) |
| 28 | *C.* goose genotype I | 2 | 2 | 2 | Geese (1), Peafowl (1) |
| 29 | *C.* goose genotype II | 1 | 1 | 1 | Swan (1) |
| 30 | *C.* ground squirrel genotype II | 1 | 2 | 2 | Marmot (1), Squirrel (1) |
| 31 | *C.* hamster genotype | 1 | 2 | 1 | Hamster (2) |
| 32 | *C.* horse genotype | 6 | 7 | 5 | Donkeys (2), Hedgehogs (1), Horses (2), Marmot (1), Squirrel (1) |
| 33 | *C. homai* | 4 | 6 | 2 | Guinea pig (5), Mice (1) |
| 34 | *C. hominis* | 14 | 15 | 11 | Bats (2), Calves (1), Camels (1), Captive mammals (1), Donkeys (2), Dugong (1), Horses (1), Macaques (2), Monkeys (1), non-human primates (2), Wild animals (1) |
| 35 | *C. macropodum* | 1 | 1 | 1 | Wild animals (1) |
| 36 | *C.* marmot genotype I | 1 | 1 | 1 | Marmot (1) |
| 37 | *C.* marmot genotype II | 1 | 1 | 1 | Marmot (1) |
| 38 | *C. meleagridis* | 13 | 13 | 4 | Birds (3), **Chickens (7)**, Foxes (2), Minks (1) |
| 39 | *C. microti* | 1 | 1 |  | Voles (1) |
| 40 | *C.* mink genotype | 5 | 5 | 2 | Donkeys (1), Minks (4) |
| 41 | *C.* monkey genotype | 1 | 1 | 1 | Macaque (1) |
| 42 | *C.* mongolian gazelle genotype | 1 | 1 | 1 | Mongolian Gazelles (1) |
| 43 | *C. muris* | 19 | 26 | 17 | Birds (2), Cats (1), Captive mammals (1), Deer (1), Dogs (1), Goats (3), Guinea pigs (1), Hamsters (1), Mice (3), Monkeys (1), non-human primates (1), Orangutans (1), **Rats (6)**, Sheep (1), Wild animals (1), Wild rodents and insectivore (1) |
| 44 | *C.* NEV 10 | 1 | 1 | 1 | Tigers (1) |
| 45 | *C. occultus* | 10 | 13 | 6 | Alpacas (1), Calves (1), Camels (2), Cattle (1), **Rats (7)**, Mica/hamster/mice/rats (1) |
| 46 | *C. parvum* | **93** | **112** | 37 | Alpacas (1), Apes (1), Bats (2), Birds (4), **Calves (25)**, Camels (2), Cats (4), Cattle (9), Chickens (2), Chinchillas (2), Cows (1), Crows (1), Deer (3), Dogs (4), Donkeys (2), Goats (5), Horses (2), Lambs (1), Lions (1), Lizards (1), Mice (3), Minks (1), Monkeys (4), non-human primates (1), Pigeons (1), Pigs (2), Racoons (1), Rats (7), Sheep (7), Shrew (1), Squirrels (2), Swan (1), Takins (1), Tigers (3), Voles (2), Wild animals (1), Yaks (1) |
| 47 | *C.* pika genotype | 1 | 1 | 1 | Pikas (1) |
| 48 | *C. proventriculi* | 2 | 2 | 2 | Birds (1), Coot (1) |
| 49 | *C.* rat genotype I | 3 | 3 | 2 | Rats (2), Squirrels (1) |
| 50 | *C.* rat genotype II | 3 | 3 | 2 | Mice (1), Squirrels (2) |
| 51 | *C.* rat genotype III | 4 | 8 | 3 | Mica/hamster/mice/rats (1), Mice (1), **Rats (6)** |
| 52 | *C.* rat genotype IV | 9 | 15 | 6 | Calves (1), Cats (1), Dogs (1), Mica/hamster/mice/rats (1), **Rats (10)**, Shrews (1) |
| 53 | *C.* ratti | 2 | 2 | 2 | Mica/hamster/mice/rats (1), Shrews (1) |
| 54 | *C. rubeyi* | 1 | 2 | 2 | Marmot (1), Squirrels (1) |
| 55 | *C. ryanae* | **40** | **47** | 10 | Artiodactyla (1), Buffaloes (2), **Calves (20)**, Cats (1), Cattle (17), Deer (1), Donkeys (1), Goats (1), Sheep (1), Yaks (2) |
| 56 | *C. ryanae* cattle type | 1 | 4 | 1 | Yaks (4) |
| 57 | *C. ryanae* buffalo type | 1 | 2 | 1 | Yaks (2) |
| 58 | *C. sciurinum* | 1 | 1 | 1 | Mice (1) |
| 59 | *C. scrofarum* | 8 | 11 |  | Boars (3), **Pigs (8)** |
| 60 | *C. serpentis* | 4 | 10 | 4 | Bats (2), Calves (1), **Snakes (6)**, Geckos/Lizards/Snakes (1) |
| 61 | *C.* skunk genotype | 2 | 3 | 2 | Racoons (2), Shrews (1) |
| 62 | *C. struthionis* | 1 | 1 | 1 | Calves (1) |
| 63 | *C. suis* | 13 | 13 | 7 | Deer (1), Dogs (1), Donkeys (1), Goats (1), **Pigs (7)**, Rats (1), Voles (1), |
| 64 | *C.* SW1 genotype | 1 | 1 | 1 | Voles (1) |
| 65 | *C. ubiquitum* | **33** | **43** | 21 | Alpacas (1), Antelope (1), Artiodactyla (1), Birds (1), Calves (1), Camels (1), Cattle (1), Chinchillas (2), Deer (3), Dogs (1), Donkeys (1), Goats (4), Hedgehogs (1), Mica/Hamster/Mice/Rats (1), Mice (1), Monkeys (1), Rats (3), **Sheep (12)**, Squirrel (3), Voles (2), Yaks (1) |
| 66 | *C. varanii* | 2 | 5 | 2 | Lizards (2), Snakes (4) |
| 67 | *C. viatorum* | 7 | 9 | 4 | Mica/Hamster/Mice/Rats (1), Mice (2), **Rats (5),** Shrews (1) |
| 68 | *C.* vole genotype I | 1 | 1 | 1 | Voles (1) |
| 69 | *C.* vole genotype II | 1 | 1 | 1 | Voles (1) |
| 70 | *C.* vole genotype V | 1 | 1 | 1 | Voles (1) |
| 71 | *C. xiaoi* | 21 | 36 | 6 | Antelope (1), Deer (2), Cattle (1), Goats (13), Peafowl (1), **Sheep (18)** |
| 72 | *C. tyzzeri* | 5 | 5 | 2 | Mice (3), Rats (2), |
| 73 | *C. wrairi* | 3 | 4 | 2 | Guinea pig (3), Squirrels (1) |
| 74 | *C.* W25 | 1 | 1 | 1 | Voles (1) |
| Genotype like | *C. galli* like | 1 | 1 | 1 | Birds (1) |
| Genotype like | *C.* NEV 10 like | 1 | 5 | 3 | Lions (2), Lynx (1), Tigers (2) |
| Genotype like | *C. suis* like | 3 | 4 | 3 | Cattle (2), Rats (1), Yaks (1) |
| Genotype like | *C. mortiferum* like | 1 | 1 | 1 | Rats (1) |
| Genotype like | *C. xiaoi* like | 1 | 1 | 1 | Chickens (1) |
| Mixed | *C. parvum + C. andersoni* | 1 | 1 | 1 | Cattle (1) |
| Mixed | *C. homai + C. hominis* | 1 | 1 | 1 | Guinea pigs (1) |
| Mixed | *C. bovis + C. ryanae* | 2 | 3 | 2 | Calves (1), Cattle (2) |
| Mixed | *C. bovis + C. andersoni* | 1 | 1 | 1 | Cattle (1) |
| Mixed | *C. bovis + C. parvum* | 3 | 3 | 3 | Calves (1), Cattle (1), Goats (1) |
| Mixed | *C. parvum* + *C. ryanae* | 1 | 1 | 1 | Cattle (1) |
| Mixed | *C. parvum + C. ryanae + C. andersoni* | 1 | 1 | 1 | Cattle (1) |
| Mixed | *C. parvum + C. bovis + C. ryanae* | 1 | 1 | 1 | Cattle (1) |

*Other *Cryptosporidium* taxa identified included *Cryptosporidium* spp. – Rats (GenBank accession no. MT561515.1; n = 1), *Cryptosporidium* spp. – Voles (KY644567; n=1), *Cryptosporidium* spp. novel 2 – Rats & Mice (n = 2), *Cryptosporidium* sp. novel 1 – Squirrel (n = 1), a novel *Cryptosporidium* genotype – Squirrel & Voles (n = 2), and a new genotype *Cryptosporidium* spp. KSFM – Wild Rodents (n = 1). *C.: Cryptosporidium*. Bold numbers refer to the most frequent species/genotype per source. Host names were arranged in alphabetical order. The highest frequencies of hosts are in bold text.
